# Association of gamma-glutamyl transferase with subclinical coronary atherosclerosis and cardiac outcomes in non-alcoholics

**DOI:** 10.1038/s41598-020-75078-6

**Published:** 2020-10-22

**Authors:** Yong-Giun Kim, Gyung-Min Park, Seung Bum Lee, Dong Hyun Yang, Joon-Won Kang, Tae-Hwan Lim, Hong-Kyu Kim, Jaewon Choe, Seung-Whan Lee, Young-Hak Kim

**Affiliations:** 1 Department of Cardiology, Ulsan University Hospital, University of Ulsan College of Medicine, 877, Bangeojinsunhwan-doro, Dong-gu, Ulsan, 44033 Republic of Korea; 2 Department of Gastroenterology and Hepatology, Ulsan University Hospital, University of Ulsan College of Medicine, 877 Bangeojinsunhwando-ro, Dong-gu, Ulsan, 44033 Republic of Korea; 3 Department of Radiology, Asan Medical Center, University of Ulsan College of Medicine, Seoul, Republic of Korea; 4 Department of Health Screening and Promotion Center, Asan Medical Center, University of Ulsan College of Medicine, Seoul, Republic of Korea; 5 Department of Cardiology, Asan Medical Center, University of Ulsan College of Medicine, Seoul, Republic of Korea

**Keywords:** Cardiology, Gastroenterology

## Abstract

In an asymptomatic population, we determined the relationship between serum gamma-glutamyl transferase (GGT) and subclinical atherosclerosis, using coronary computed tomography angiography (CCTA). This was a retrospective observational cohort study which analyzed 5120 consecutive asymptomatic individuals with no prior history of coronary artery disease or significant alcohol intake who voluntarily underwent CCTA as part of a general health examination. All subjects were stratified into tertiles based on GGT levels. Degree and extent of subclinical coronary atherosclerosis were evaluated using CCTA. Cardiac events were a composite of all-cause death, myocardial infarction, unstable angina, and coronary revascularization. After adjustment for cardiovascular risk factors, there were no significant differences among GGT tertiles in terms of adjusted odds ratios for non-calcified and mixed plaques. The risk of any atherosclerotic and calcified plaques, significant stenosis, multi-vessel disease, and significant stenosis in the left main or proximal left anterior descending artery was higher in the third GGT tertile than in the first tertile (all *p* < 0.05). Over a median 5.4-year follow-up, the third GGT tertile had significant adjusted hazards ratios for cardiac events than did the first GGT tertile, even after stepwise adjustment for cardiovascular risk factors (all *p* < 0.01). In asymptomatic individuals, elevated GGT was independently associated with high-risk feature atherosclerosis and poorer cardiac outcomes.

## Introduction

Gamma-glutamyl transferase (GGT) is a hepatobiliary enzyme synthesized in epithelial cells of the intrahepatic duct. Elevated GGT is a specific indicator of obstructive biliary diseases and excessive alcohol intake^1^. An association between serum GGT and nonalcoholic fatty liver disease (NAFLD) has been established, and some authors consider serum GGT as a surrogate marker of NAFLD^2,3^. Several studies have reported that serum GGT levels are related to cardiovascular diseases (CVD)^4–6^. Based on studies to date, it may be assumed that GGT represents a link between NAFLD and atherosclerosis, with insulin resistance as a common pathophysiology^7–9^. Recently, coronary computed tomography angiography (CCTA) has proven to be effective in providing a comprehensive evaluation of coronary atherosclerosis^10^. An association of elevated serum GGT and subclinical coronary atherosclerosis detected by CCTA has also been reported^11,12^. However, there are limited data on clinical outcomes including future cardiac events. Therefore, the present study sought to evaluate the relationship between serum GGT and subclinical coronary atherosclerosis as well as cardiac events in a large cohort of asymptomatic Korean individuals who voluntarily underwent CCTA for early detection of coronary artery disease (CAD).

## Results

### Baseline characteristics

All subjects were classified into tertiles based on GGT levels (Tertile 1 ≤ 15 IU/L, n = 1618; Tertile 2, 16–27 IU/L, n = 1791; Tertile 3, ≥ 28 IU/L, n = 1711). The baseline characteristics of 5120 study participants according to the tertiles of GGT levels are summarized in Table 1. The mean age of study participants was 53.8 ± 7.6 years, and 3486 (68.1%) were males. The prevalence of males, diabetes mellitus, hypertension, hyperlipidemia, current smoking, and obesity was significantly higher in tertiles of increasing GGT levels. In addition, BMI, waist circumference, systolic and diastolic blood pressure, as well as levels of fasting blood glucose, total cholesterol, LDL cholesterol, triglyceride, uric acid, AST, and ALT increased with GGT category. In contrast, levels of HDL cholesterol were lower in tertiles of decreasing GGT levels.

**Table 1:** Baseline characteristics of the study population according to the tertiles of gamma-glutamyl transferase.

Characteristics	Overall (n = 5,120)	Gamma-glutamyl transferase			
		Tertile 1 ≤ 15 IU/L (n = 1618)	Tertile 2 16–27 IU/L (n = 1791)	Tertile 3 ≥ 28 IU/L (n = 1711)	*p* value
Age, years	53 (49–58)	53 (49–59)	54 (50–59)	53 (49–57)	< 0.001
Male sex, no. (%)	3486 (68.1)	618 (38.2)	1364 (76.2)	1504 (87.9)	< 0.001
Body mass index, kg/m^2^	24.3 (22.5–26.2)	23.0 (21.2–24.7)	24.5 (22.8–26.2)	25.5 (23.8–27.2)	< 0.001
Waist circumference, cm	85 (80–90)	80 (75–86)	86 (80–90)	89 (84–93)	< 0.001
Systolic blood pressure, mmHg	119 (110–128)	115 (107–124)	119 (110–128)	122 (113–130)	< 0.001
Diastolic blood pressure, mmHg	76 (69–83)	72 (65–79)	76 (69–83)	79 (73–86)	< 0.001
Diabetes mellitus, no. (%)	772 (15.1)	147 (9.1)	280 (15.6)	345 (20.2)	< 0.001
Hypertension, no. (%)	1778 (34.7)	406 (25.1)	634 (35.4)	738 (43.1)	< 0.001
Hyperlipidemia, no. (%)	1559 (30.4)	358 (22.1)	555 (31.0)	646 (37.8)	< 0.001
Current smoker, no. (%)	1024 (20.0)	136 (8.4)	371 (20.7)	517 (30.2)	< 0.001
Obesity, no. (%)	2114 (41.3)	362 (22.4)	756 (42.3)	996 (58.2)	< 0.001
Family history of coronary artery disease^a^, no. (%)	788 (15.4)	252 (15.6)	282 (15.7)	254 (14.8)	0.739
Fasting blood glucose, mg/dL	99 (93–108)	96 (91–102)	100 (94–109)	103 (96–114)	< 0.001
Total cholesterol, mg/dL	193 (172–217)	190 (171–214)	192 (171–214)	198 (176–221)	< 0.001
Low-density lipoprotein cholesterol, mg/dL	120 (101–120)	117 (99–136)	121 (102–140)	123 (104–143)	< 0.001
High-density lipoprotein cholesterol, mg/dL	51 (43–61)	57 (48–68)	49 (43–60)	48 (41–56)	< 0.001
Triglycerides, mg/dL	108 (78–156)	84 (64–113)	109 (81–151)	138 (100–201)	< 0.001
Creatinine, mg/dL	0.9 (0.8–1.0)	0.8 (0.7–0.9)	0.9 (0.8–1.0)	0.9 (0.9–1.0)	< 0.001
Uric acid, mg/dL	5.5 (4.5–6.4)	4.7 (3.9–5.6)	5.6 (4.8–6.4)	6.0 (5.2–7.0)	< 0.001
Aspartate aminotransferase, IU/L	25 (21–31)	22 (19–26)	24 (21–29)	28 (24–36)	< 0.001
Alanine aminotransferase, IU/L	22 (16–31)	17 (13–21)	22 (17–29)	30 (22–43)	< 0.001
High-sensitivity C-reactive protein ≥ 2 mg/L	47 (0.9)	7 (0.4)	21 (1.2)	19 (1.1)	0.046

Values are presented as median (interquartile range) or number (%).

Pearson's chi-square test or Fisher's exact test were used for categorical variables and one-way analysis of variance or Kruskal–Wallis test were used for numerical variables.

^a^Coronary artery disease in a first-degree relative of any age.

### CCTA findings

CCTA findings according to GGT tertiles are showed in Table 2. Mean CACS was 38.2 ± 136.4. Among the study participants, 405 (7.9%) had significant stenosis of coronary artery (≥ 50% stenosis diameter) in at least one coronary artery on CCTA. Mean CACS increased with GGT category (*p* < 0.001). A significant difference was presented in the prevalence of calcified, non-calcified, mixed, or any atherosclerotic plaques according to GGT level (all *p* < 0.001). Scores which reflect plaque burden on CCTA such as segment involvement score, segment stenosis score, and modified Duke prognostic score increased with GGT tertile (all *p* < 0.001). Furthermore, the prevalence of significant coronary artery stenosis, multi-vessel disease, and significant stenosis in the left main (LM) or proximal left anterior descending (LAD) artery also increased according to GGT tertile (all *p* < 0.001).

**Table 2:** Comparison of coronary computed tomography angiographic findings according to the tertiles of gamma-glutamyltransferase.

Variables	Overall	Gamma-glutamyl transferase			*p* value
		Tertile 1	Tertile 2	Tertile 3	
Mean coronary artery calcium score	38.2 ± 136.4	20.8 ± 74.9	42.6 ± 152.2	50.0 ± 160.3	< 0.001
Any atherosclerotic plaque, no. (%)	2083 (40.7)	471 (29.1)	778 (43.4)	834 (48.7)	< 0.001
**Plaque characteristics, no. (%)**					
Calcified plaque	1371 (26.8)	300 (18.5)	521 (29.1)	550 (32.1)	< 0.001
Non-calcified plaque	940 (18.4)	214 (13.2)	346 (19.3)	380 (22.2)	< 0.001
Mixed plaque	451 (8.8)	91 (5.6)	164 (9.2)	196 (11.5)	< 0.001
Segment involvement score	1.0 ± 1.7	0.7 ± 1.4	1.1 ± 1.8	1.3 ± 1.9	< 0.001
Segment stenosis score	0.6 ± 1.9	0.3 ± 1.2	0.7 ± 2.0	0.8 ± 2.3	< 0.001
Modified Duke prognostic score	1.2 ± 0.6	1.1 ± 0.4	1.2 ± 0.7	1.2 ± 0.7	< 0.001
**Number of stenosed coronary arteries, no. (%)**					
Significant stenosis	405 (7.9)	78 (4.8)	146 (8.2)	181 (10.6)	< 0.001
One-vessel disease	298 (5.8)	67 (4.1)	102 (5.7)	129 (7.5)	< 0.001
Multi-vessel disease	107 (2.1)	11 (0.7)	44 (2.5)	52 (3.0)	< 0.001
Left main or proximal left anterior descending artery	136 (2.7)	21 (1.3)	54 (3.0)	61 (3.6)	< 0.001

Values are presented as mean ± standard deviation or number (%).

Pearson's chi-square test or Fisher's exact test were used for categorical variables and one-way analysis of variance or Kruskal–Wallis test were used for numerical variables.

### Association between GGT levels and subclinical atherosclerosis

The association between GGT levels and subclinical atherosclerosis is described in Table 3. Univariable analyses revealed that increasing tertiles of GGT were significantly associated with subclinical coronary atherosclerosis detected by CCTA. After adjustment for cardiovascular risk factors (age, sex, obesity, diabetes mellitus, hypertension, hyperlipidemia, current smoking, family history of CAD, and hs-CRP), no statistically significant differences were observed in the adjusted odds ratios (OR) for non-calcified and mixed plaques between GGT tertiles. The risk of atherosclerotic (OR 1.25, 95% confidence interval [CI] 1.04 − 1.50, *p* = 0.016) and calcified plaques (OR 1.24, 95% CI: 1.02 − 1.52, *p* = 0.033), significant stenosis (OR 1.51, 95% CI: 1.10 − 2.07, *p* = 0.011), multi-vessel disease (OR 2.47, 95% CI: 1.22 − 4.98, *p* = 0.012), and significant stenosis in the LM or proximal LAD artery (OR 1.94, 95% CI: 1.11 − 3.38, *p* = 0.020) was higher in the third GGT tertile than in the first GGT tertile.

**Table 3:** Association between gamma-glutamyl transferase levels and coronary computed tomography angiographic findings.

Variables	Univariable		Multivariable	
	Odds ratio (95% CI)	*p* value	Odds ratio (95% CI)	*p* value
**Any atherosclerotic plaque**				
Tertile 1 (reference)	1	–	1	–
Tertile 2	1.87 (1.62–2.16)	< 0.001	1.09 (0.92–1.29)	0.348
Tertile 3	2.32 (2.01–2.67)	< 0.001	1.25 (1.04–1.50)	0.016
**Calcified plaque**				
Tertile 1 (reference)	1	–	1	–
Tertile 2	1.80 (1.53–2.12)	< 0.001	1.12 (0.93–1.35)	0.255
Tertile 3	2.08 (1.77–2.45)	< 0.001	1.24 (1.02–1.52)	0.033
**Non-calcified plaque**				
Tertile 1 (reference)	1	–	1	–
Tertile 2	1.57 (1.31–1.89)	< 0.001	1.06 (0.87–1.30)	0.560
Tertile 3	1.87 (1.56–2.25)	< 0.001	1.20 (0.97–1.48)	0.099
**Mixed plaque**				
Tertile 1 (reference)	1	–	1	–
Tertile 2	1.69 (1.30–2.21)	< 0.001	1.00 (0.75–1.33)	0.997
Tertile 3	2.17 (1.68–2.81)	< 0.001	1.17 (0.87–1.58)	0.306
**Significant stenosis**				
Tertile 1 (reference)	1	–	1	–
Tertile 2	1.75 (1.32–2.33)	< 0.001	1.15 (0.85–1.56)	0.370
Tertile 3	2.34 (1.78–3.07)	< 0.001	1.51 (1.10–2.07)	0.011
**Multi-vessel disease**				
Tertile 1 (reference)	1	–	1	–
Tertile 2	3.68 (1.89–7.15)	< 0.001	1.99 (1.00–3.95)	0.051
Tertile 3	4.58 (2.38–8.81)	< 0.001	2.47 (1.22–4.98)	0.012
**Significant stenosis in the left main or proximal left anterior descending artery**				
Tertile 1 (reference)	1	–	1	–
Tertile 2	2.36 (1.42–3.93)	0.001	1.63 (0.95–2.78)	0.074
Tertile 3	2.81 (1.70–4.64)	< 0.001	1.94 (1.11–3.38)	0.020

*CI* confidence interval.

Covariates in the multivariable model include age, sex, obesity, diabetes mellitus, hypertension, hyperlipidemia, creatinine, uric acid, current smoking, family history of coronary artery disease, and high-sensitivity C-reactive protein ≥ 2 mg/L.

### Clinical outcomes

During the follow-up period (median 5.4 years [interquartile range, 4.4–6.3 years]), a total of 165 cardiac events occurred in 154 patients: 52 all-cause deaths, three myocardial infarctions, 10 unstable angina requiring hospitalization, and 100 coronary revascularizations (Table 4). After adjustment for stepwise cardiovascular risk factors, the third GGT tertile had a significant adjusted hazard ratios for cardiac event, which was defined as a composite of all-cause death, myocardial infarction, unstable angina requiring hospitalization, or coronary revascularization, relative to that of the first GGT tertile (all *p* < 0.05, Table 5).

**Table 4:** Clinical outcomes according to the tertiles of gamma-glutamyl transferase.

	Tertile 1 (n = 1618)	Tertile 2 (n = 1791)	Tertile 3 (n = 1711)	*p* value*
**Cardiac event, no. (%)**				
Death/myocardial infarction/unstable angina requiring hospitalization/coronary revascularization	30 (1.9)	57 (3.2)	67 (3.9)	0.002
**Clinical event, no. (%)**				
Death	20 (1.2)	17 (0.9)	15 (0.9)	0.510
Myocardial infarction	1 (0.1)	2 (0.1)	0 (0)	0.396
Unstable angina requiring hospitalization	0 (0)	1 (0.1)	9 (0.5)	0.001
Coronary revascularization	10 (0.6)	38 (2.1)	52 (3.0)	< 0.001
Percutaneous coronary intervention	10 (0.6)	37 (2.1)	49 (2.9)	
Coronary artery bypass surgery	0 (0)	1 (0.1)	3 (0.2)	
Death/myocardial infarction/unstable angina requiring hospitalization	21 (1.3)	19 (1.1)	24 (1.4)	0.624

Values are presented as n (%). *p* values were calculated using the log-rank test*.

**Table 5:** Univariable and multivariable analyses of gamma-glutamyl transferase levels for cardiac events, corrected for clinical risk factors.

Clinical outcomes	Unadjusted		Model 1		Model 2		Model 3	
	HR (95% CI)	*p* value	HR (95% CI)	*p* value	HR (95% CI)	*p* value	HR (95% CI)	*p* value
**Cardiac events**								
Tertile 1 (reference)	1	–	1	–	1	–	1	–
Tertile 2	1.71 (1.10–2.66)	0.017	1.39 (0.88–2.20)	0.155	1.34 (0.85–2.12)	0.207	1.35 (0.86–2.12)	0.192
Tertile 3	2.13 (1.38–3.27)	0.001	1.99 (1.26–3.14)	0.003	1.84 (1.16–2.91)	0.010	1.85 (1.17–2.92)	0.008

*HR* hazards ratio; *CI* confidence interval.

Model 1 was adjusted for age and sex; model 2 was adjusted further for diabetes mellitus, hypertension, hyperlipidemia, and current smoking; and model 3 was adjusted further for body mass index (kg/m^2^), creatinine, uric acid, family history of coronary artery disease, and high-sensitivity C-reactive protein ≥ 2 mg/L.

## Discussion

The main findings of the present study were as follows: (1) in asymptomatic individuals, elevated serum GGT levels were significantly associated with atherosclerotic plaques, especially calcified plaques, even after adjustment for cardiovascular risk factors; (2) high serum GGT levels were an independent predictor of significant coronary atherosclerosis such as significant stenosis in at least one coronary artery, multi-vessel disease, and significant stenosis in the LM or proximal LAD; (3) during a follow-up of median 5.4 years, individuals with high serum GGT levels experienced more cardiac events.

The association between elevated GGT levels and risk of CAD or CVD has been suggested in large epidemiologic studies^4–6^. Subsequent studies have reported that higher GGT levels were significantly associated with endothelial dysfunction, carotid artery plaques, and arterial stiffness^13–15^. Moreover, recent studies have indicated that GGT levels were significantly related to high CACS and its progression, which reflects total atherosclerotic burden and is a surrogate marker of future CAD events^11,12^. However, the absence of coronary calcification does not exclude the presence of clinically significant and potentially vulnerable atherosclerotic plaques^16,17^. Although CCTA provides more comprehensive information regarding coronary atherosclerosis, there are limited data on the association between GGT and subclinical coronary atherosclerosis evaluated by CCTA. An observational cohort study with CCTA investigated the association between GGT levels and coronary atherosclerotic plaques^18^. However, it analyzed limited data without clinical outcomes. Therefore, the present study aimed to evaluate the influence of GGT levels on the risk of subclinical coronary atherosclerosis on CCTA and cardiac outcomes.

In the present study, individuals with higher GGT levels had a higher prevalence, extent, and severity of coronary atherosclerosis detected by CCTA. Even after adjustments for clinical and laboratory variables, higher GGT levels were an independent predictor of coronary atherosclerotic plaques, especially calcified plaques. Thus, our results were consistent with previous studies evaluating CACS^11,12^. Notably, higher GGT levels were an independent risk factor for high-risk feature CAD such as multi-vessel disease and significant stenosis in the LM or proximal LAD, which are known to be associated with poorer prognosis^19^. As a result, in the current study, individuals with higher GGT levels experienced more cardiac events. Therefore, our findings suggest that subjects with high serum GGT levels may benefit from preemptive cardiovascular risk evaluation and potentially, regular cardiovascular risk surveillance to guard against future cardiovascular events.

Although the exact mechanisms underpinning the association between serum GGT and CVD remain unclear, one potential pathophysiology is that they may be linked via NAFLD. Clinically, GGT is a hepatic enzyme that reflects the degree of hepatic inflammation or fibrosis^20^ and has been suggested as a surrogate marker for NAFLD^2,3^. NAFLD is regarded as the hepatic manifestation of metabolic syndrome and insulin resistance, which may increase the risk of atherosclerosis^7,8,21^. A chronic activation of systemic and hepatic inflammation may also act as a common pathway for NAFLD and CVD^22^. NAFLD is progressive across simple fatty livers to steatohepatitis, fibrosis, and finally liver cirrhosis. In particular, steatohepatitis with the potential of hepatic inflammation or fibrosis has been reported to increase the risk of cardiovascular mortality and morbidity^23–25^. To diagnose NAFLD, a liver biopsy is required which has several limitations including invasiveness, complications, sampling variability, and cost^26,27^. Ultrasonography typically usually employed as a substitute for liver biopsy, but there is a limit to the distinction between simple fatty liver and steatohepatitis^28,29^. Instead, noninvasive methods to determine the presence of hepatic inflammation or fibrosis have been developed. Of these, NAFLD fibrosis score (NFS) and Fibrosis-4 (FIB-4) are representative scoring systems that have been well-validated^30,31^. NFS include factors representing metabolic syndrome such as BMI or impaired glucose tolerance/diabetes mellitus, while FIB-4 includes age and serum markers. Although several studies have presented a significant association between NFS or FIB-4 and the prevalence or future risk of CVD, the results were not fully adjusted for other cardiovascular risk factors^32–34^. This study sought to evaluate the relationship between GGT levels as a noninvasive marker for hepatic inflammation or fibrosis and characteristics of atherosclerosis with their prognosis in a large cohort of asymptomatic Korean individuals who voluntarily underwent CCTA, which has proven to be effective in providing a comprehensive evaluation of coronary atherosclerosis^10^.

Our study has several limitations. First, the present study was performed at a single center, and all study participants voluntarily visited the hospital for a general health examination. Therefore, there was a potential of selection bias. Second, our study participants were exclusively Korean, limiting the applicability of our findings to other ethnic groups. Third, as the current study was a retrospective cohort study, these data may not fully reflect patient outcomes. Additionally, we did not specify the cause of death. Fourth, calcified plaques and higher CACS may lead to overestimation of significant coronary artery stenosis. Finally, CCTA has potential drawbacks, including radiation hazards, use of contrast, and higher cost. Therefore, although this study enrolled only volunteers, the use of CCTA in asymptomatic individuals cannot be justified.

In conclusion, this large retrospective cohort study in asymptomatic individuals undergoing CCTA demonstrated that elevated serum GGT levels were independently associated with high-risk subclinical atherosclerosis, resulting in poorer cardiac outcomes. Therefore, cardiovascular risk evaluation and surveillance should be considered in subjects with high GGT levels to prevent future cardiovascular disease. These findings should be further investigated and validated in future studies.

## Materials and methods

### Study population

**Figure 1:**
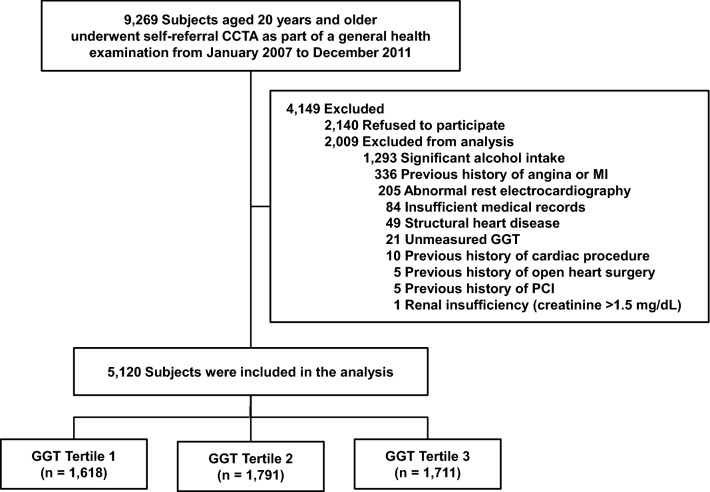
Overview of the study population. *CCTA* coronary computed tomographic angiography; *GGT* gamma-glutamyl transferase; *MI* myocardial infarction; *PCI* percutaneous coronary intervention.

This was a retrospective observational cohort study which analyzed 9269 consecutive South Korean individuals aged ≥ 20 years who had undergone self-referred CCTA evaluation as part of a general health examination at the Health Screening and Promotion Center in the Asan Medical Center between January 2007 and December 2011. Among these, 7129 (76.9%) agreed to participate in the present study. Possible risks associated with CCTA were explained, and written informed consent was obtained from each participant. Exclusion criteria included subjects with (1) a previous history of significant alcohol intake with ≥ 210 g/week in males and 140 g/week in females^35,36^; (2) a previous history of angina or myocardial infarction; (3) abnormal resting electrocardiography results, i.e., pathological Q waves, ischemic ST segments or T-wave changes, or left bundle-branch blocks; (4) insufficient medical records; (5) structural heart disease; (6) unmeasured GGT; (7) a previous cardiac procedure; (8) a previous history of open-heart surgery or percutaneous coronary intervention; or (9) renal insufficiency (creatinine > 1.5 mg/dL). A final total of 5120 subjects were enrolled (Fig. 1). The study was approved by the local Institutional Review Board of the Asan Medical Center, Seoul, Korea (2016–1068). Written informed consent under the 'Ethics, consent and permissions' was obtained from each participant. This study was carried out in accordance with Good Clinical Practice (GCP) guidelines and the Declaration of Helsinki.

### Clinical and laboratory measurements

Basic demographic data of study participants were acquired from a database maintained by the Health Screening and Promotion Center at the Asan Medical Center. Medical history including angina, myocardial infarction, stroke, structural heart disease, open heart surgery, percutaneous coronary intervention, previous cardiac procedures, diabetes mellitus, hypertension, hyperlipidemia, or smoking status, was obtained from the responses in the systemized self-report questionnaire issued prior to the general health examination^37^.

Height and weight were obtained with subjects wearing light clothing and no shoes. Body mass index (BMI) was calculated as weight in kilograms divided by the square of height in meters (kg/m^2^). Waist circumference (cm) was measured mid-way between the lower costal margin and iliac crest at the end of a normal expiration of breath by a well-trained nurse. Blood pressure was measured on the right arm after a ≥ 5 min rest using an automatic manometer and an appropriate cuff size. After overnight fasting, early morning blood samples were drawn from the antecubital vein into vacuum tubes and subsequently analyzed in the central certified laboratory of the Asan Medical Center. The concentrations of GGT, aspartate aminotransferase (AST), alanine aminotransferase (ALT), fasting plasma glucose, creatinine, uric acid, fasting total cholesterol, triglycerides, high-density lipoprotein (HDL) cholesterol, low-density lipoprotein (LDL) cholesterol, and high-sensitivity C-reactive protein (hs-CRP) were measured^38^.

Obesity was defined as a BMI ≥ 25 kg/m^2^ on the basis of an Asian-specific cutoff point as recommended in the World Health Organization. Diabetes mellitus was defined as subjects with a self-reported history of diabetes and/or treatment with dietary modification, use of anti-diabetic medication as indicated on the systemized questionnaire, or those with a fasting plasma glucose ≥ 126 mg/dL. Hypertension was defined as blood pressure ≥ 140/90 mmHg or a self-reported history of hypertension and/or use of anti-hypertensive medication. Hyperlipidemia was defined as total cholesterol ≥ 240 mg/dL or use of an anti-hyperlipidemic medication. A family history of CAD was defined as having a first-degree relative of any age with CAD based on the self-report questionnaire^37^.

### CCTA image acquisition and analysis

CCTA was conducted using either single-source 64-slice CT (LightSpeed VCT, GE, Milwaukee, WI, USA) or dual-source CT (Somatom Definition, Siemens, Erlangen, Germany). A standard scanning protocol was used, as previously described^10^. All CCTA scans were analyzed using a dedicated workstation (Advantage Workstation, GE; or Volume Wizard, Siemens) by experienced cardiovascular radiologists (DHY, JWK, and THL). According to the guidelines of the Society of Cardiovascular Computed Tomography, a 16-segment coronary artery tree model was used^39^. A coronary artery calcium score (CACS) was measured and categorized by scores of 0, 1 to 10, 11 to 100, 101 to 400, and > 400^40^. Plaques containing calcified tissue involving more than 50% of the plaque area (density > 130 HU) were classified as calcified, plaques with < 50% calcium were classified as mixed, and plaques without calcium were classified as non-calcified lesions^41^. The contrast-enhanced portion of the coronary lumen was semi-automatically traced at the site of maximal stenosis and compared with the mean value of the proximal and distal reference sites. Stenosis ≥ 50% was defined as significant^10^. The overall plaque burden was determined from coronary artery plaque scores calculated from modified Duke prognostic scores, segment stenosis scores, and segment involvement scores, as described previously^42^.

### Clinical outcomes

Follow-up clinical data were obtained by a review of medical records or telephone interviews using trained personnel through to the end of June 2017. A cardiac event was defined as a composite of all-cause death, myocardial infarction, unstable angina requiring hospitalization, or coronary revascularization. The diagnosis of myocardial infarction was based on the presence of new Q waves in at least two contiguous leads, or an elevation of creatine kinase or its myocardial band isoenzyme to at least three times the upper limit of the normal range at follow-up. Revascularization was performed if there was a stenosis of at least 50% of the diameter noted on invasive coronary angiography with a positive stress test result or if there was a stenosis of at least 70% observed on invasive coronary angiography^43^.

### Statistical analysis

Categorical variables are expressed as frequencies with percentages. Continuous variables are expressed as the mean and standard deviation. Between-group comparisons were performed using Pearson's chi-square test or Fisher's exact test for categorical variables and one-way analysis of variance or Kruskal–Wallis test for numerical variables, as appropriate. Univariable and multivariable analyses were performed using a logistic regression model to analyze the association between serum GGT levels and subclinical coronary atherosclerosis on CCTA. Based on previous epidemiologic studies^44,45^, we selected covariates in the multivariable model according to their clinical importance and statistical significance, which included age, sex, obesity, diabetes mellitus, hypertension, hyperlipidemia, creatinine, uric acid, current smoking, family history of CAD, and hs-CRP ≥ 2 mg/L. Unadjusted and adjusted odds ratios with 95% confidence intervals for the logistic regression were calculated. To investigate the associations of serum GGT levels and cardiac events, we also conducted the Cox proportional hazards regression analyses with adjustment for potential confounders. Stepwise multivariable models were determined by the backward variable selection approach. All reported *p* values are two-sided, and *p* < 0.05 was considered statistically significant. Data manipulation and statistical analyses were performed using SPSS software (Version 18; SPSS Inc., Chicago, IL, USA).

## Data Availability

The datasets used and/or analyzed during the current study available from the corresponding author on request.

## References

[1] Whitfield JB (2001). Gamma glutamyl transferase. Crit. Rev. Clin. Lab. Sci..

[2] Angulo P (2002). Nonalcoholic fatty liver disease. N. Engl. J. Med..

[3] Hossain IA, Rahman SMM, Rahman MK, Ali L (2016). Gamma glutamyl transferase is an independent determinant for the association of insulin resistance with nonalcoholic fatty liver disease in Bangladeshi adults: Association of GGT and HOMA-IR with NAFLD. Diabetes Metab. Syndr..

[4] Lee DS (2007). Gamma glutamyl transferase and metabolic syndrome, cardiovascular disease, and mortality risk: the Framingham heart study. Arterioscler. Thromb. Vasc. Biol..

[5] Strasak AM (2008). Longitudinal change in serum gamma-glutamyltransferase and cardiovascular disease mortality: a prospective population-based study in 76,113 Austrian adults. Arterioscler. Thromb. Vasc. Biol..

[6] Wannamethee G, Ebrahim S, Shaper AG (1995). Gamma-glutamyltransferase: determinants and association with mortality from ischemic heart disease and all causes. Am. J. Epidemiol..

[7] Kozakova M (2012). Fatty liver index, gamma-glutamyltransferase, and early carotid plaques. Hepatology.

[8] Ndrepepa G, Colleran R, Kastrati A (2018). Gamma-glutamyl transferase and the risk of atherosclerosis and coronary heart disease. Clin. Chim. Acta..

[9] Targher G, Byrne CD (2015). Circulating markers of liver function and cardiovascular disease risk. Arterioscler. Thromb. Vasc. Biol..

[10] Park GM (2015). Prevalence of coronary atherosclerosis in an Asian population: findings from coronary computed tomographic angiography. Int. J. Cardiovasc. Imaging..

[11] Cho YK (2015). Association between serum gamma-glutamyltransferase and the progression of coronary artery calcification. Atherosclerosis.

[12] Atar AI, Yilmaz OC, Akin K, Selcoki Y, Er O, Eryonucu B (2013). Association between gamma-glutamyltransferase and coronary artery calcification. Int. J. Cardiol..

[13] Arinc H (2013). Serum gamma glutamyl transferase and alanine transaminase concentrations predict endothelial dysfunction in patients with non-alcoholic steatohepatitis. Ups. J. Med. Sci..

[14] Toshikuni N, Asaji T, Nakanishi Y, Nagasawa SY, Uenishi H, Tsutsumi M (2015). Elevated serum gamma-glutamyl transpeptidase levels and fatty liver strongly predict the presence of carotid plaque. J. Atheroscler. Thromb..

[15] Jung CH (2011). Serum gamma-glutamyltransferase is associated with arterial stiffness in healthy individuals. Clin. Endocrinol. (Oxf.).

[16] van Velzen JE (2011). Comparison of the relation between the calcium score and plaque characteristics in patients with acute coronary syndrome versus patients with stable coronary artery disease, assessed by computed tomography angiography and virtual histology intravascular ultrasound. Am. J. Cardiol..

[17] Yoon YE (2012). The absence of coronary artery calcification does not rule out the presence of significant coronary artery disease in Asian patients with acute chest pain. Int. J. Cardiovasc. Imaging..

[18] Cho HS (2015). Clinical significance of serum bilirubin and gamma-glutamyltransferase levels on coronary atherosclerosis assessed by multidetector computed tomography. Nutr. Metab. Cardiovasc. Dis..

[19] Min JK (2012). All-cause mortality benefit of coronary revascularization vs. medical therapy in patients without known coronary artery disease undergoing coronary computed tomographic angiography: results from CONFIRM (COronary CT angiography evaluation for clinical outcomes: an international multicenter registry). Eur. Heart J..

[20] Korkmaz H, Unler GK, Gokturk HS, Schmidt WE, Kebapcilar L (2015). Noninvasive estimation of disease activity and liver fibrosis in nonalcoholic fatty liver disease using anthropometric and biochemical characteristics, including insulin, insulin resistance, and 13C-methionine breath test. Eur. J. Gastroenterol. Hepatol..

[21] Loria P, Lonardo A, Targher G (2008). Is liver fat detrimental to vessels?: intersections in the pathogenesis of NAFLD and atherosclerosis. Clin. Sci. (Lond.).

[22] Schindhelm RK (2007). Alanine aminotransferase predicts coronary heart disease events: a 10-year follow-up of the Hoorn Study. Atherosclerosis..

[23] Ekstedt M (2006). Long-term follow-up of patients with NAFLD and elevated liver enzymes. Hepatology.

[24] Kim D, Kim WR, Kim HJ, Therneau TM (2013). Association between noninvasive fibrosis markers and mortality among adults with nonalcoholic fatty liver disease in the United States. Hepatology.

[25] Chen Y (2015). Advanced fibrosis associates with atherosclerosis in subjects with nonalcoholic fatty liver disease. Atherosclerosis..

[26] Bedossa P, Carrat F (2009). Liver biopsy: the best, not the gold standard. J. Hepatol..

[27] Emanuele E (2008). Is biopsy always necessary? Toward a clinico-laboratory approach for diagnosing nonalcoholic steatohepatitis in obesity. Hepatology.

[28] Charatcharoenwitthaya P, Lindor KD (2007). Role of radiologic modalities in the management of non-alcoholic steatohepatitis. Clin. Liver Dis..

[29] Li Q, Dhyani M, Grajo JR, Sirlin C, Samir AE (2018). Current status of imaging in nonalcoholic fatty liver disease. World J. Hepatol..

[30] Angulo P (2007). The NAFLD fibrosis score: a noninvasive system that identifies liver fibrosis in patients with NAFLD. Hepatology.

[31] Sterling RK (2006). Development of a simple noninvasive index to predict significant fibrosis in patients with HIV/HCV coinfection. Hepatology.

[32] Onnerhag K, Hartman H, Nilsson PM, Lindgren S (2019). Non-invasive fibrosis scoring systems can predict future metabolic complications and overall mortality in non-alcoholic fatty liver disease (NAFLD). Scand. J. Gastroenterol..

[33] Parikh NS, VanWagner LB, Elkind MSV, Gutierrez J (2019). Association between nonalcoholic fatty liver disease with advanced fibrosis and stroke. J. Neurol. Sci..

[34] Song DS, Chang UI, Kang SG, Song SW, Yang JM (2019). Noninvasive serum fibrosis markers are associated with coronary artery calcification in patients with nonalcoholic fatty liver disease. Gut Liver.

[35] Chalasani N (2012). The diagnosis and management of non-alcoholic fatty liver disease: practice guideline by the American Association for the Study of Liver Diseases, American College of Gastroenterology, and the American Gastroenterological Association. Hepatology.

[36] O'Shea RS, Dasarathy S, McCullough AJ (2010). Practice guideline Committee of the American Association for the Study of Liver D, Practice Parameters Committee of the American College of G alcoholic liver disease. Hepatology.

[37] Park GM (2014). Model for assessing cardiovascular risk in a Korean population. Circ. Cardiovasc. Qual. Outcomes..

[38] Park GM (2016). Family history of diabetes and the risk of subclinical atherosclerosis. Diabetes Metab..

[39] Raff GL (2009). SCCT guidelines for the interpretation and reporting of coronary computed tomographic angiography. J. Cardiovasc. Comput. Tomogr..

[40] Agatston AS, Janowitz WR, Hildner FJ, Zusmer NR, Viamonte M, Detrano R (1990). Quantification of coronary artery calcium using ultrafast computed tomography. J. Am. Coll. Cardiol..

[41] Leber AW (2006). Accuracy of 64-slice computed tomography to classify and quantify plaque volumes in the proximal coronary system: a comparative study using intravascular ultrasound. J. Am. Coll. Cardiol..

[42] Min JK (2007). Prognostic value of multidetector coronary computed tomographic angiography for prediction of all-cause mortality. J. Am. Coll. Cardiol..

[43] Group BDS (2009). A randomized trial of therapies for type 2 diabetes and coronary artery disease. N. Engl. J. Med..

[44] Goff DC (2014). 2013 ACC/AHA guideline on the assessment of cardiovascular risk: a report of the American College of Cardiology/American Heart Association Task Force on practice guidelines. J. Am. Coll. Cardiol..

[45] Conroy RM (2003). Estimation of ten-year risk of fatal cardiovascular disease in Europe: the SCORE project. Eur. Heart J..

